# Dental Implants with a Calcium Ions-Modified Surface and Platelet Concentrates for the Rehabilitation of Medically Compromised Patients: A Retrospective Study with 5-Year Follow-Up

**DOI:** 10.3390/ma14112718

**Published:** 2021-05-21

**Authors:** Marco Mozzati, Giorgia Gallesio, Giulio Menicucci, Carlo Manzella, Margherita Tumedei, Massimo Del Fabbro

**Affiliations:** 1 Private Practitioner, SIOM Oral Surgery and Implantology Center, 10126 Turin, Italy; marcomozzati@siompoliambulatorio.it (M.M.); giorgiagallesio@siompoliambulatorio.it (G.G.); 2 Prosthodontic Department, School of Dentistry, University of Turin, 10124 Turin, Italy; giuliomenicucci@libero.it (G.M.); carlo.manzella@studiosphera.it (C.M.); 3 Department of Medical, Oral and Biotechnological Sciences, University of Chieti, 66100 Chieti, Italy; margherita.tumedei@unich.it; 4 Department of Biomedical, Surgical and Dental Sciences, University of Milano, 20122 Milan, Italy; 5 IRCCS Orthopedic Institute Galeazzi, Dental Clinic, 20161 Milan, Italy

**Keywords:** calcium-ion modified surface, dental implants, growth factors, medically compromised patients, plasma rich in growth factors, platelets, systemic diseases, osteoporosis, diabetes, rheumatoid arthritis, lupus erythematosus systemic

## Abstract

Background: Platelet concentrates are biological, autologous products obtained from the patient’s whole blood, consisting of a supraphysiological concentration of platelets and growth factors, that have proved beneficial in different applications in the medical and dental fields. They are used in several medical and dental applications to enhance tissue healing. Previous evidence shows that platelet concentrates may be beneficial in patients with compromised systemic conditions, in which the healing process is impaired. Aim: To evaluate the 5-year clinical outcome of implant treatment using acid-etched implants with calcium ions-modified surface in association with plasma rich in growth factors, in patients with systemic diseases of a different nature. Methods: Charts of 99 medically compromised patients, who had received a total of 224 dental implants from January 2013 to June 2013, were retrospectively evaluated. Patients were divided into four groups, according to their condition: diabetes (n = 39 patients), osteoporosis (n = 36), lupus erythematosus systemic (n = 5), rheumatoid arthritis (n = 19). The main outcomes were implant survival, marginal bone level (MBL) change and complications throughout follow-up. Results: Mean follow-up was 63.06 ± 1.90 months (range 60.1 to 66.4 months). In total, eight implants failed in 6 diabetic patients and 4 in 3 patients with rheumatoid arthritis. Overall 5-year implant survival was 94.6%. In total, 30 complications occurred in 24 patients, mostly transient, and no severe adverse event occurred. Overall MBL change was 0.45 ± 0.12 mm, with no significant differences among groups. Conclusions: In the present sample of medically compromised patients, rehabilitation with calcium ions-modified surface implants associated with plasma rich in growth factors proved to be a safe and effective treatment. The satisfactory results achieved after 5-year follow-up are comparable to those historically reported for healthy patients.

## 1. Introduction

The use of platelet concentrates in oral surgery and implantology guarantees a whole series of benefits, both when they are interposed between soft tissues and bone, and when they are used as grafting material in bone gaps or as a filler in simple extractions without performing flaps. A number of studies and systematic reviews reported that the use of blood components in oral surgery allows for faster epithelialization, pain reduction, better healing and tissue formation [1,2]. The adhesive and hemostatic properties of the blood components can be particularly useful in different situations. For example in the case of extensive exposure of deep connective tissue, in patients with coagulation deficiency, with systemic diseases (e.g., diabetes, chronic renal failure, autoimmune diseases), or undergoing treatment with steroids, chemotherapy, radiotherapy in the head and neck area, or bisphosphonates. In fact, all these conditions compromise the ability to heal properly, and often lead to postoperative complications. For example, poor wound healing has been a common finding in diabetic patients and is characterized by a decrease in the amount of wound collagen and lowered tensile strength. The risks of complications increase with the duration of hyperglycemia because the production of advanced glycation end-products (i.e., modifications of plasma proteins or lipids that become nonenzymatically glycated and oxidized after contact with aldose sugars, and accumulate in the blood vessel wall, perturbing cell function and structure [3]) causes micro- and macrovascular complications and increases susceptibility to infection, vascular changes, and impaired healing [4]. Additionally, biological drugs such as cytokines and monoclonal antibodies, that are now being used in the treatment of autoimmune diseases, such as rheumatoid arthritis, can affect wound healing. Although these biological drugs are effective for many diseases, some of them can also cause adverse reactions, such as decreased immune function and opportunistic infections. For example, animal studies have shown that wound healing is inhibited by tumor necrosis factor-α (TNF-α) inhibitors [5]. In regards to wound healing in patients with rheumatoid arthritis, impaired wound healing has been reported with TNF-α inhibitors [6]. In addition, corticosteroids therapy directly inhibits the production and activity of osteoclasts, osteoblasts, and osteocytes. In particular, stimulation of osteocyte apoptosis leads to osteonecrosis [7]. Because of these healing problems, patients with systemic diseases are often excluded from implant therapy.

Osseointegration has been defined by Albrektsson et al. in 1981 as “the direct structural and functional connection between living bone and the surface of a load-bearing artificial implant” [8]. In order to make implant osseointegration more and more predictable, allowing to extend implant therapy to those patients with healing impairments, over the years researchers aimed at improving implant surface topography and chemistry, in order to enhance cell attachment and bone formation. To achieve effective and durable osseointegration there is a need for interfaces that not only achieve good bone integration, but also substantially limit and reduce bacterial colonization [9,10]. In the last years, the addition of calcium ions at titanium implant surfaces has been reported to promote surface-based provisional matrix formation, platelet activation, and osseointegration [11], and is also responsible for the synthesis and binding of several bone-related proteins. Additionally, less bacterial adhesion and biofilm formation using calcium-ion modified implant surfaces, with respect to microrough surfaces, was reported [12].

The rationale of using platelet concentrates stands on the assumption that the presence of growth factors can represent an additional stimulation for tissue healing in these patients contrasting the inhibition produced by their metabolic disease, in order to improve bone and soft tissue healing [13]. In 1999, Anitua first introduced a new protocol for platelet gel preparation, producing the so-called plasma rich in growth factors (PRGF)-Endoret^®^ [14] and then described its advantages and technical application in many different kinds of surgical procedures. Though the efficacy of platelet-rich preparations in promoting bone healing and regeneration has been at the center of an academic debate for many years [15,16], they seem to offer many advantages. They provide elements that are essential for tissue regeneration, such as signalling molecules and scaffolding materials. In fact, platelet concentrates represent a source of multiple active growth factors (the main ones being platelet-derived growth factor, transforming growth factor β, vascular endothelial growth factor, epidermal growth factor, insulin-like growth factor, and fibroblast growth factor), able to promote wound healing and increase tissue vascularization [17]. There is evidence that platelet concentrates may reduce local inflammation, postoperative pain, and symptoms after oral surgery procedures [18,19]. They also showed antimicrobial properties, which may help in controlling post-operative infection [20,21]. The use of PRGF-Endoret^®^ can accelerate bone regeneration after tooth extraction [22], and around implants [23].

Previous studies suggested that platelet concentrates represent a helpful adjunctive tool for promoting tissue healing in medically compromised patients. For example, they may bring advantages for both prevention and treatment of medication-related osteonecrosis of the jaw (MRONJ), a condition often afflicting patients with cancer or osteoporosis, under antiresorptive or antiangiogenic drugs for their primary disease [24,25,26].

Given the above premises, a retrospective study was planned with the aim of evaluating the outcome of implant therapy after at least 5 years follow-up, in patients with different types of systemic diseases who were treated using acid-etched titanium implants with calcium ions-modified surface, associated with plasma rich in growth factors.

## 2. Materials and Methods

All patients were treated in a private practice setting, in compliance with the principles laid down in the Declaration of Helsinki on medical research protocols. Institutional review board approval of the IRCCS Orthopedic Institute Galeazzi was obtained for retrospective studies on implants (Prot. No. 75/2019-L2058). The study sample was drawn from the population of patients with metabolic disease who had received dental implants from January 2013 to June 2013.

Inclusion criteria were: presence of a systemic disease like diabetes, osteoporosis or rheumatoid arthritis; need for implant-supported rehabilitation; a minimum follow-up period of 5 years after surgery. Before implant placement, all patients were extensively informed of the risk of complications related to their metabolic disease and of the other treatment alternatives for rehabilitation. All subjects included in the study gave written, informed consent to the treatment, and agreed to be available for follow-up clinical visits, including postoperative radiographs, all of which was carefully documented. Exclusion criteria were: uncontrolled coagulation disorders, acute myocardial infarction within the past 6 months, radiotherapy to head or neck within the past 12 months.

### 2.1. Plasma Rich in Growth Factors

For preparing the PRGF-Endoret^®^ (from Endogenous Regenerative Therapy) the patient’s blood is collected in 9 mL tubes containing 0.2 mL of 3.8% trisodium citrate (BTI Biotechnology Institute, Álava, Spain) as anticoagulant. The blood volume drawn is minimal, up to 40 mL for complex interventions or large defects that need multiple applications. PRGF preparation requires a single centrifugation at 460 g for 8 min (PRGF^®^ System, BTI Biotechnology Institute, Álava, Spain). Erythrocytes and the buffy coat layer are deposited at the bottom of the tube, and the upper part is a plasma component, which does not contain leukocytes and pro-inflammatory cytokines. Such components are manually divided in to two fractions. The lower fraction of about 2 mL, just above the buffy coat, is the PRGF; the upper portion is the plasma poor in growth factors (PPGF). The PRGF typically contains two- to three-fold the baseline value of platelet concentration. PRGF is activated before application using 20 μL of calcium chloride (BTI Biotechnology Institute, Álava, Spain)/mL PRGF [27], in order to induce platelet degranulation and growth factors release. Different final products can be obtained with PRGF: liquid, gel, membrane, fibrin clot. PRGF membranes are obtained by activating the liquid deposited on a flat surface, they, therefore, have the same composition as the clot.

### 2.2. Implant Characteristics

All the implants used in this study had a special surface (UnicCa^®^, BTI Biotechnology Institute, Álava, Spain), consisting of a calcium ions deposition over the implants that have a surface with a differential roughness (optima^®^ surface, BTI Biotechnology Institute, Álava, Spain) [12,28]. In particular, the surface roughness varies in each area of the implant: in the coronal area (implant neck) surface roughness is attenuated, in the threads the roughness is high and in the valley is medium, in order to adapt to the different surrounding tissues. The variations in roughness among implant neck, threads, and valleys are obtained by differential acid etching treatment. The whole surface is then further chemically modified with calcium ions. This provides implants with an electropositive, clean, and active surface that mantains superhydrophilic properties. As a consequence, the surface promptly interacts with the growth factors released by the PRGF [12,28]. The calcium ions, which have fundamental functions in bone regeneration process, are released by the implant surface in two phases. The first phase takes place at the implantation phase, and lasts for a few minutes, promoting coagulation on the implant surface, platelet adhesion, activation and release of growth factors [11,28]. Procoagulant properties allow to immediately fill the gaps around the implant with a coagulum. The formation of the matrix in the implant–bone gap allows fast implant stabilization. In the second phase, the release is prolonged up to several weeks, and mantains a concentration of calcium ions around the implant able to promote a successful osseointegration [11,12]. The calcium ions embedded to the implant surface and slowly released up to almost 3 months [11], in fact work as a platelet-activating factor, allowing the surface to become a growth factors emitter over time. Another interesting feature of the UnicCa^®^ surface is that it may have a bacteriostatic action [12], potentiated by the PRGF [20,21], which may reduce the formation of microbial biofilms, reducing the risk of peri-implantitis.

### 2.3. Surgical Procedures

A professional oral hygiene session was performed on each patient one week before surgery. All patients received antibiotic prophylaxis with amoxicillin (Pfizer Italia s.r.l., Milano, Italy) 1 g every 12 h from the day before surgery and for 5 days thereafter. Before surgery and before the administration of local anesthesia (4% of articaine chlorhydrate and epinephrine 1:100,000) (Pierrel S.p.A., Capua, Italy), 5 to 20 mL of peripheral blood, depending on the type of surgery, were drawn. The same operator performed all implant surgeries. To enhance implant osseointegration and both hard and soft tissues healing PRGF-Endoret^®^ (BTI^®^, Biotechnology Institute, Vitoria, Alava, Spain) was always associated to implant surgery in such patients following the protocol described by Anitua et al. Before installation, all implants (BTI^®^, Biotechnology Institute) were carefully embedded in liquid plasma rich in growth factors with the aim of bioactivating the implant surface. A portion of the PRGF clot could also be flattened and used as a covering membrane before flap closure. Primary closure of the surgical site was attained using a resorbable 4-0 vicryl suture (Ethicon, Inc., Somerville, NJ, USA). Patients were recommended to strictly follow proper oral hygiene procedures (gently brushing with a soft toothbrush and rinsing with 0.2% chlorhexidine solution twice daily for 14 days), and a soft food diet for one week. Sutures were removed 14 days after surgery. Standard prosthetic procedures occurred three to four months after implant placement. All patients were recalled for the usual follow-up, which consisted of a yearly clinical and radiological examination.

### 2.4. Outcome Variables

Primary variables were:

1. Implant survival at 5-year follow-up. The implant survival criteria were: each implant had to be immobile at clinical test evaluation and connected to a fixed prostheses providing functional service, with no sign or symptoms of pain or infection and no peri-implant radiolucency in periapical radiographic images;

2. Marginal bone loss after 5-year follow-up. Bone level changes were evaluated on periapical radiographs, through the software ImageJ version 1.46 (National Institutes of Health, Bethesda, MD, USA). The known implant length and diameter were used for calibration. The linear distance parallel to the implant axis between the implant platform and the most coronal bone-to-implant contact point, as identifiable on the radiographs, was taken at mesial and distal aspects. Measurements were taken at insertion (baseline), at the prosthesis delivery, and at the 5-year follow-up. The difference between any follow-up measurement and the baseline values at both mesial and distal aspects represented the marginal bone level change. Mesial and distal measurements were averaged so as to have a single value per implant;

3. Biological complications (e.g., peri-implant mucositis, peri-implantitis, dehiscence) or mechanical complications (e.g., screw loosening or fracture, prosthesis fracture) that occurred during the follow-up period, and required the operator’s intervention to be fixed.

Secondary variables were:

Full-mouth plaque score (FMPS), measured as the percentage of sites with plaque over the total number of sites (considering 4 sites evaluated per each implant/tooth present);

Full-mouth bleeding scores (FMBS), measured as the percentage of sites bleeding on gentle probing (considering 4 sites evaluated per each implant/tooth present).

### 2.5. Statistical Analysis

Statistical analysis was undertaken using GraphPad Prism 5.03 (GraphPad Software, Inc., La Jolla, CA, USA). Quantitative data were presented as mean values ± 1 standard deviation. Qualitative data were presented as absolute values and percentages. Proportion of failures, complications, and marginal bone level change in the different groups of patients were compared using Friedman test for qualitative data or analysis of variance for quantitative data. Significance level was set at *p* = 0.05.

## 3. Results

This article was reported following the strengthening the reporting of observational studies in epidemiology (STROBE) guidelines (http://www.strobe-statement.org, accessed on 22 April 2021). Following the selection criteria, 99 consecutive patients were included (45 males and 54 females). The mean age at surgery was 55.42 ± 9.55 years (range 37 to 71 years). Table 1 reports the main features of patients, divided by systemic disease. There were 22 smokers (of which 8 smoked > 10 cigarettes/day). FMPS averaged 30.22 ± 7.69% (range 16% to 53%) and FMBS averaged 29.94 ± 6.89% (range 13% to 55%). Overall, 57 patients suffered from periodontitis, and 20 patients had parafunction. Mean follow-up was 63.06 ± 1.90 months (range 60.1 to 66.4 months).

The total n. of implants inserted was 224. Overall, 49 implants (21.9%) were inserted with the platform at crestal level, and 175 (78.1%) at subcrestal level. Implant distribution in the arches is detailed in Table 2. Table 3 shows the length and diameter distribution of the implants used.

Prosthesis type was screwed in 184 cases (82.1%) and cemented in 40 cases (17.9%). Prosthesis material was ceramic in 71 cases (31.7%) and zirconia in 153 cases (68.3%). Occlusal antagonist was ceramic in 61 cases (27.2%) and natural teeth in 163 cases (72.8%). 

**Figure 1:**
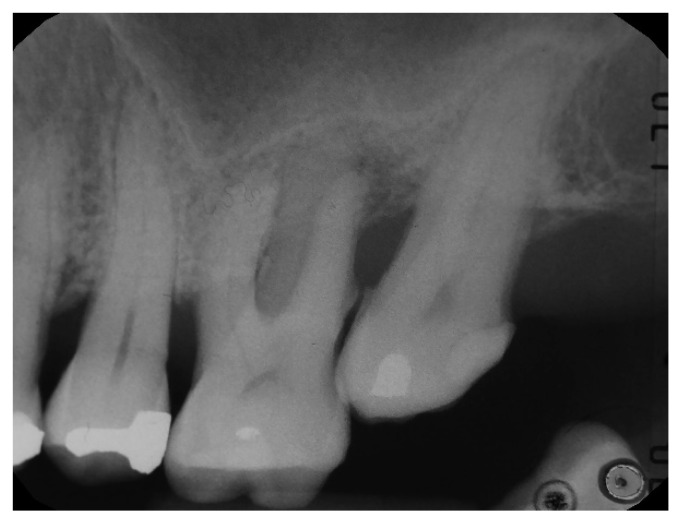
Patient in therapy with oral bisphosphonates for osteoporosis. Initial intraoral radiography.

**Figure 2:**
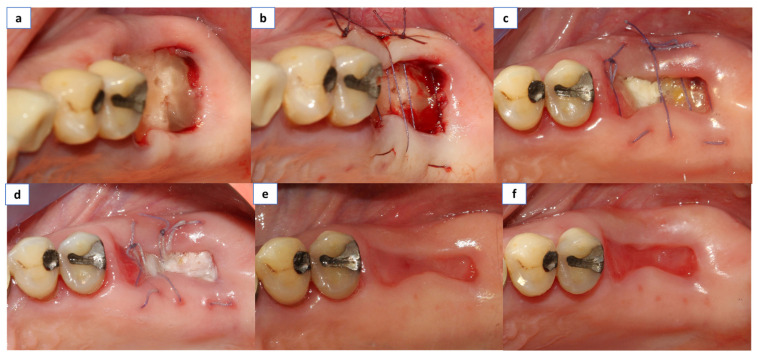
(**a**) The PRGF was applied within the socket and the membrane was laid above within the socket wall, (**b**) Suturing was done with resorbable material in all cases (Vycril_ 4/0, Ethicon, Inc., Somerville, New Jersey, US), (**c**) Clinical control at 3 days, (**d**) Clinical control at 7 days, and (**e**) Clinical control at 14 days. (**f**) Clinical control at 21 days.

**Figure 3:**
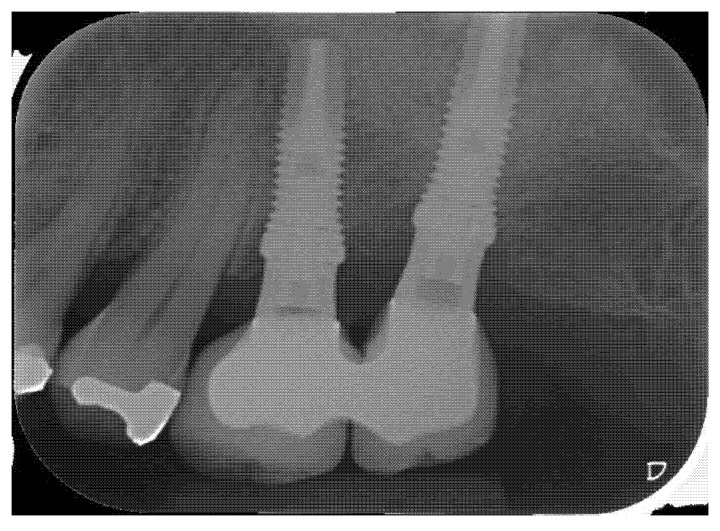
Radiographic control at 4 months.

**Figure 4:**
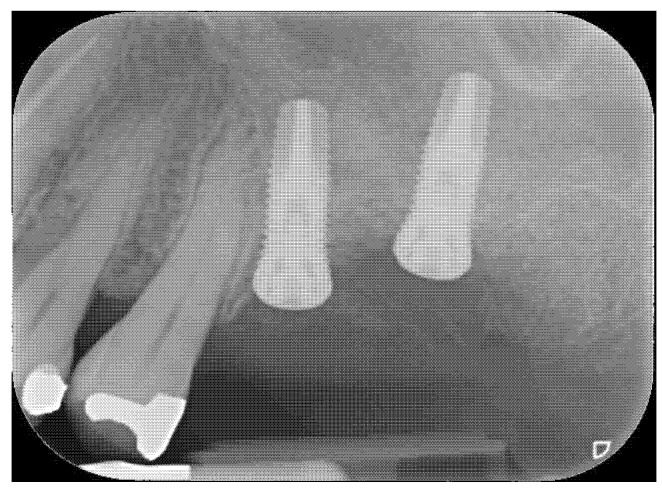
Radiographic control at 5 years.

Table 4 shows the main results, divided by systemic disease. In total, 12 implants (5.4%) failed in nine patients (9.1%) within two months of placement. Eight failures occurred in 6 diabetic patients, and 4 failures occurred in 3 patients with rheumatoid arthritis. All patients experiencing failure were smokers, and 7 out of 9 were also affected by periodontitis. No further failure occurred throughout the follow-up. There were 30 complications in 24 patients (13 hematoma, 9 post-surgical bleeding, 6 disesthesia, 2 paresthesia). The status of peri-implant soft tissues was healthy in 189 cases (84.4%), while 23 cases of mucositis (10.3%) were recorded. In 11 cases (5.3%) the soft tissue condition was not reported. There were 22 dehiscences (9.8%). Mean bone loss was 0.45 ± 0.14 mm (range 0.16–0.99 mm). Figure 1, Figure 2, Figure 3 and Figure 4 show a clinical case with a radiographic control up to 5 years follow-up.

## 4. Discussion

The aim of the present study was to evaluate the performance of dental implants having calcium ions-modified surface, associated with platelet concentrates, in patients with systemic disease. Very few publications on the specific commercial products used in this study are present in the literature, outside the developer’s ones, and no 5-year reports on such implant surface in medically compromised patients are available. The findings of the present study showed that for a total of 224 implants inserted, the implant survival rate can be as high as 94.6% after at least 5-year follow-up.

Several studies and systematic reviews of the literature evaluated the outcome of implant treatment in medically compromised patients [29,30,31,32,33,34,35]. These studies highlighted that there are very few absolute medical contraindications to dental implant placement. Nevertheless, many conditions may pose an increased risk for implant failure or complications. In Table 5 are resumed the main conditions that have been identified to increase the risk for implant treatment success [29,30,31,32,34,35,36].

In general, when the medical condition can be controlled by proper lifestyle, or pharmacological therapy, the implant treatment outcomes tend to normalize, becoming similar to those of medically healthy patients. It seems that the nature of the disorder itself, whether acquired or congenital, is not as determinant to the treatment success as the degree of disease control is [30].

In diabetic patients with elevated glycemic levels, the risk of numerous systemic co-morbidities, including periodontal disease, infection, impaired wound healing, and peri-implant complications are increased [29,32,37,38].

Previous studies [39] documented a poor relationship between glycemic levels and implant-related clinical complications for type 2 diabetes patients having mean glycemic levels (HbA1c) between 6.3% and 13.1% over two years after implant placement, and above 8% at the time of implant placement [40]. Oates et al. [40] in a review study reported after two years a survival rate of 96.2%, comparable with previous reports in non-diabetic subjects. A more recent systematic review concluded that patients with poorly controlled diabetes may present impaired osseointegration, higher risk of peri-implantitis, and higher level of implant loss, as compared to non-diabetic patients [41]. On the other hand, when diabetes is well controlled, implant therapy is predictable and safe, with a complication rate similar to that of healthy subjects [41]. A recent study, on a limited population, concluded that among medically compromised conditions evaluated (diabetes, osteoporosis, hypothyroidism), the highest implant survival rates are found among diabetic patients [35].

In the present study, 8 implants failed in 6 out of 39 diabetic patients. All patients experiencing failure showed additional risk factors. In fact, they were also smokers and affected by periodontal disease, and all failures occurred within two months of placement, suggesting that diabetes alone probably was not the main determinant for failure. Therefore, in line with the current literature [42], the present data suggest that, in the absence of multiple risk factors, diabetic patients receiving PRGF may display a highly effective healing process. Other studies on dental implants in diabetic patients showed that platelet concentrates may prove advantageous and safe for both the patient and the operator by reducing post-operative discomfort, such as edema, swelling, hematoma and post-surgical bleeding, and enhancing wound healing [43].

Patients affected by lupus and rheumatoid arthritis suffer from inflammation, swelling, and pain in and around the joints and other body organs. Slow-healing wounds, including leg and foot ulcers, are a known complication of several autoimmune inflammatory diseases, including RA, lupus, and scleroderma [34]. For many people, these wounds can take months or even years to heal. Anti-inflammatory treatment of RA with glucocorticoids (GC) or NSAIDs are supposed to negatively influence bone metabolism and healing. Furthermore, RA itself is suspected to promote bone healing complications. However, a recent prospective controlled study suggested that another autoimmune inflammatory disease, lichen planus, is not a prominent local player in the genesis of implant failure [44]. A recent review focused on the outcome of dental implants in patients with autoimmune or muco-cutaneous diseases (oral lichen planus, pemphigus, epidermolysis bullosa, Sjögren´s syndrome, systemic lupus erythematosus, or systemic sclerosis) found relatively high implant survival rates in patients affected by these diseases. However, the results derived by studies with low evidence level such as case reports and case series. Therefore, the review concluded that, in spite the implant survival rates were comparable to those of healthy patients, no guidelines exist, and no conclusions can be drawn regarding implant treatment of such patients, for which frequent recall is recommended [34]. In the present paper, there were no failures in the 12 implants placed in patients with lupus. Conversely, of the 40 implants placed in 19 patients with rheumatoid arthritis, four failed within two months of placement. Three implants were lost in two smokers, and one in a periodontal patient, again suggesting that the simultaneous presence of multiple risk factors might have a prominent role in implant failure.

Regarding patients affected by osteoporosis, a recent systematic review with meta-analysis found no significant difference in implant survival between patients with and without osteoporosis, but the former displayed a higher marginal bone loss around implants [36]. Osteoporosis per se might not represent a risk for implant survival, but it is well-known that in patients taking antiresorptive drugs, there is an increased risk for osteonecrosis of the jaws, especially following interventions involving jawbone, like tooth extraction or implant surgery [45]. A large clinical study on 235 osteoporotic women under bisphosphonates undergoing implant treatment associated with plasma rich in growth factors, showed 98.7% of implant survival, and no cases of osteonecrosis after 5 years follow-up [46]. Among the possible etiological factors of MRONJ, microbial infection has been suggested [12,45]. The surface features of the implants used in the present study, together with the use of a platelet concentrate, may counteract such risk factor. As reported by a recent study, the attenuated roughness of the implants in the coronal area, along with the use of PRGF-Endoret^®^, significantly reduces the bacterial colonization [28,47].

Previous studies showed that plasma rich in growth factors may stimulate faster epithelization, reduce postoperative pain and symptoms, and enhance hard and soft tissue healing in a number of oral surgery procedures in both healthy and medically compromised subjects [1,2,14,17,18,19,24,25,26,42,46,48,49,50,51]. Favero et al. in a study on animal model of implant osseointegration, reported that the percentages of new bone increased by 4 times after 2 weeks at the UnicCa^®^ surface, and higher new bone percentages were found at BTI UnicCa^®^, which immediately initiates the regenerative process and reduces implant failure [52,53]. Though, in spite of convincing preclinical evidence, the actual benefit of the adjunctive use of platelet concentrates in implant treatment needs to be supported by further evidence-based, long-term clinical studies. Nevertheless, the use of any procedures that might enhance and support hard and soft tissue healing, and possibly reduce postsurgical adverse events and complications, in the medically compromised patients, should be recommended. The role of supportive dental care and the frequency of recalls in these patients also need to be explored and clarified in future studies.

The main limitation of the present study is the absence of a control group composed by medically compromised patients in which standard implants instead of modified UnicCa^®^ surface implants were used in combination with PRGF, or by patients treated without using a platelet concentrate, or by systemically healthy patients. However, given the retrospective nature of the study, this was not feasible. Another limitation was that the groups were not balanced in size. This was due to the fact that some conditions such as diabetes and osteoporosis are more frequent than others, and we considered medically compromised patients consecutively treated in a given time period.

## 5. Conclusions

Oral rehabilitation using calcium ions-modified surface implants associated with plasma rich in growth factors proved to be a feasible option, leading to satisfying outcomes, in a group of medically compromised patients over a five-year follow-up period. The compromised patient needs special attention, and any approach aimed at reducing discomfort, pain, treatment time, and improving patient’s quality of life, as well as increasing healing predictability, must be pursued. Further studies are needed to confirm the promising results of the present study.

## Figures and Tables

**Table 1:** Features of patients in the different groups.

Variable	Diabetes	Rheumatoid Arthritis	Osteoporosis	Systemic Lupus Erythematosus
N (%)	39 (39.39%)	19 (19.19%)	36 (36.36%)	5 (5.05%)
FMBS (%)	34.18 ± 7.10%	26.73 ± 7.03%	27.94 ± 7.41%	29.00 ± 2.55%
FMPS (%)	30.87 ± 7.96%	29.0 ± 6.69%	29.31 ± 6.14%	30.80 ± 3.77%
Periodontitis (%)	27 (69.23%)	8 (42.10%)	20 (55.55%)	2 (40%)
Parafunction (%)	7 (17.94%)	3 (15.79%)	8 (22.22%)	2 (40%)
Gender (M/F)	35/4	1/18	5/31	3/2
Age (mean ± SD)	50.70 ± 8.96	51.90 ± 8.13	62.18 ± 6.50	57.0 ± 10.77
Follow-up, months (mean ± SD)	62.71 ± 1.93	63.38 ± 1.87	62.95 ± 1.99	58.47 ± 1.57

N: patients number; FMBS: full mouth bleeding score; FMPS: full mouth plaque score; M/F: male/female; SD: standard deviation.

**Table 2:** Summary of the implant/site distribution.

**Implant Diameter, mm**	**Implant Length, mm**				
	7.5	8.5	10	11.5	13
3.75	0	0	18	7	4
4	0	6	49	50	6
5	2	27	31	7	0
5.5	7	9	1	0	0

**Table 3:** Summary of the implant size distribution.

Implant Distribution Per Site		
Site	n. Impl	Failed
17	5	0
16	24	2
15	25	0
14	16	0
13	0	0
23	0	0
24	17	1
25	23	1
26	18	2
27	3	0
37	5	0
36	13	1
35	17	1
34	11	2
33	2	1
43	1	0
44	8	0
45	15	1
46	17	0
47	4	0

**Table 4:** Summary of the main results after 5-year follow-up, divided by systemic disease.

Systemic Condition	n. Patients	n. Implants	n. Failed Implants (%)	Impl Survival %	n. Patients with Failure (%)	Complications (%)	Complic. in Pats (%)	Bone Loss/Impl. mm	n. Mucositis (%)	n. Dehiscences (%)
Osteoporosis	36	87	0	100%	0	11 (12.6%)	8 (22.2%)	0.45 ± 0.12	4 (4.60%)	7 (8.05%)
Diabetes	39	85	8 (9.4%)	90.6%	6 (15.4%)	10 (11.8%)	10 (25.6%)	0.45 ± 0.13	10 (11.76%)	9 (10.59)
Rheum.arthr.	19	40	4 (10%)	90%	3 (15.8%)	6 (15%)	4 (21.1%)	0.42 ± 0.15	9 (22.5%)	5 (12.5%)
Lupus	5	12	0	100%	0	3 (25%)	2 (40%)	0.49 ± 0.18	0 (0%)	1 (8.33%)
total	99	224	12 (5.4%)	94.6%	9 (9.1%)	30 (13.4%)	24 (24.2%)	0.45 ± 0.12	23 (10.3%)	22 (9.8%)

**Table 5:** Summary of previous studies reporting on implants in medically compromised patients.

First Author, Journal, Year	Medical Condition	Implant Treatment Outcome
Scully et al., 2007 [29]	Diabetes mellitus	Implant success rate: 86–96%
	Cancer	Success rate 97% (40% to 100%) in post-operative irradiated group. 100% success in non-irradiated group
Diz et al., 2013 [30]	Rheumatoid arthritis and other connective diseases	Cumulative 3-year implant success rate: 96.1%
	Immunocompromised patient HIV-positive	2 implants 6-months success rate: 100%
	Epidermolysis bollosa	102 implants, 108 months success rate: 100%
Gomez De Diego et al., 2014 [31]	Smoking	Tot implants 2.523, survival range: 93.8–97.3%
	Smoking and diabetes type I	Tot implants 720, survival rate: 98.1%
	Smoking and Osteoporosis	Tot implants 6.946, survival rate: 96.4%
Alzahrani et al., 2016 [32]	Diabete	Tot implants 1342, survival range: 86–100%
De Medeiros et al., 2017 [36]	Osteoporosis	failure rate diseased patients (n = 217): 4.70% failure rate healthy patients (n = 890): 3.57% (Risk ratio: 0.98)
Striezel et al., 2019 [34]	Oral Lichen planus	100 patients, 302 implants, survival rate: 98.3%
	Epidermolysis bollosa	27 patients, 152 implants, survival rate: 98.7%
	Sjogren syndrome	71 patients, 272 implants, survival rate: 94.2%
	Systemic sclerosis	6 patients, 44 implants, survival rate: 97.7%
	Pemphigus	2 implants, survival rate: 100%
	Lupus erythematosus	6 implants, survival rate: 100%
Parihar et al., 2020 [35]	Medically compromised (diabetes 25 patients, osteoporosis 16 pat., hypothyroidism 12 pat., organ transplant 10 pat., cardiovascular disease 5 pat.)	Healthy patients, 72 implants, 4 failures (5.56%) Medically compromised patients, 80 implants, 18 failures (22.5%)

## Data Availability

All experimental data to support the findings of this study are available contacting the corresponding author upon request.
